# The role of electrostriction in the generation of acoustic waves by optical forces in water

**DOI:** 10.1016/j.pacs.2022.100445

**Published:** 2022-12-30

**Authors:** N.G.C. Astrath, B. Anghinoni, G.A.S. Flizikowski, V.S. Zanuto, L.C. Malacarne, M.L. Baesso, T. Požar, D. Razansky

**Affiliations:** a Department of Physics, Universidade Estadual de Maringá, 87020-900 Maringá, Brazil; b Faculty of Mechanical Engineering, University of Ljubljana, 1000 Ljubljana, Slovenia; c Institute for Biomedical Engineering and Institute of Pharmacology and Toxicology, Faculty of Medicine, University of Zurich, 8057 Zurich, Switzerland; d Institute for Biomedical Engineering, Department of Information Technology and Electrical Engineering, ETH Zurich, 8093 Zurich, Switzerland

**Keywords:** Electrostriction, Radiation forces, Photo-induced lensing effect, Pressure-transients, Kerr lens

## Abstract

We present semi-analytical solutions describing the spatiotemporal distributions of temperature and pressure inside low-absorbing dielectrics excited by tightly focused laser beams. These solutions are compared to measurements in water associated with variations of the local refractive index due to acoustic waves generated by electrostriction, heat deposition, and the Kerr effect at different temperatures. The experimental results exhibited an excellent agreement with the modeling predictions, with electrostriction being the dominant transient effect in the acoustic wave generation. Measurements at $4.0{\;}^{\circ }C$ show that the thermoelastic contribution to the optical signal is significantly reduced due to the low thermal expansion coefficient of water at this temperature.

## 1 Introduction

The definitive knowledge of the electromagnetic forces acting inside matter under the influence of external fields remains an open problem in Physics. It is directly related to the century-old Abraham–Minkowski dilemma [1], [2], which deals with the electromagnetic momentum transfer in dielectric media. The topic has recently drawn significant attention in the context of applications relying on complete manipulation of optical forces [3], such as optical traps [4], photonic devices [5] and optofluidic technology [6], [7].

When electromagnetic fields are applied to continuous matter, variety of effects can take place. Regarding internal forces, one known contribution is related to the tendency of the matter to be compressed towards the regions of higher field intensity due to the interaction between the induced dipoles and the applied electromagnetic fields. Such compression forces occur even for static fields and are commonly described as electrostriction in the case of polarizable media or magnetostriction for magnetizable media. In conductors, these striction effects can slightly deform the medium, leading to observable changes in its shape [8]. In turn, dielectric media respond by increasing their local pressure or strain until the forces are completely compensated — striction forces are thus inherently transient effects. The compensation time-scale involved is the interval it takes for the generated acoustic waves to propagate over one beam width, typically about 10–100 ns [9] when tightly focused beams of light are used as the excitation source.

Striction forces appear as important contributions in opto-acoustic techniques, such as laser-induced thermal acoustics [10] and laser induced phonons [11]. Additionally, they are often related to fluid stability [12], [13] and can also provide the necessary non-linear coupling that leads to stimulated Brillouin scattering [14], [15]. Electrostriction has also been suggested as an alternative way to improve piezoelectricity in ferroelectric materials [16].

Accurate measurements of striction forces have been historically challenging since even small or moderate thermal effects usually dominate [17]. One of the well-established methods in the literature to probe these effects consists in analyzing the diffraction generated by stimulated Brillouin scattering [18], [19], [20], [21], [22]. Alternatively, in liquids the local variation of pressure alters the local index of refraction of the material, an effect that can usually be probed in weakly absorbing transparent media by optical techniques such as interferometry and photo-induced lensing [23].

On the theoretical side, striction effects are currently not very well understood. The generally adopted description relies on a phenomenological approach derived for thermodynamic equilibrium [8], [24], [25]. For static fields, good agreement with experiments has been found [26], [27]. However, as pressure and strain perturbations must propagate at sound velocity within the medium, this theory is not expected to hold for optical excitation. Very recently, the first measurement of the electrostriction force as the dominant effect at optical frequencies has been reported along with a new microscopic-scale interpretation of its origin [23].

Here, a model for laser-induced time- and space-dependent pressure and temperature distributions inside low-absorbing fluids is presented. The model is then successfully applied to delineate an experimental signal obtained via an optical photo-induced lensing (PIL) technique, stemming from the application of a tightly focused pulsed laser beam in water. The optical Kerr and thermal effects are shown to be temporally decoupled from the electrostriction effect, providing a clear observation of the later effect at optical frequencies.

## 2 Pressure and temperature changes due to radiation forces

In the experiments performed in this work, a linearly polarized laser beam in the fundamental Gaussian mode excites a low-absorbing sample. The non-uniform excitation interaction with the sample leads to an increase in the internal energy resulting in a temperature change in the sample, which alters its mass density. A pressure variation builds up with this perturbation relaxing via emission of acoustic waves. The pressure waves generated by temperature change or by radiation forces induce variations in the sample’s local refractive index, generating a phase shift and, consequently, a wavefront distortion to the probe beam passing through the sample and cuvette walls. These effects are thus responsible for the transient signal measured in the experiments.

Due to the weak light absorption by the sample, the excitation laser beam intensity distribution along its z-axis (thickness) can be assumed constant. This allows for the simplification of the problem so that the intensity I and the electromagnetic force density $f_{\mathrm{em}}$ can be written in terms of the cylindrical coordinate r and the time t only, hence implying that the pressure p(r,t) and temperature T(r,t) distributions are also sole functions of r and t. These last two distributions within water can be calculated by solving the uncoupled differential equations

(1) $$\nabla^{2}p\left(r,t\right)-\frac{1}{\nu^{2}}\partial_{t}^{2}p\left(r,t\right)=\nabla \cdot f\left(r,t\right)-\frac{\beta A_{e}}{c_{p}}\partial_{t}I\left(r,t\right)$$

and

(2) $$\rho_{m}c_{p}\partial_{t}T\left(r,t\right)-k\nabla^{2}T\left(r,t\right)=A_{e}I\left(r,t\right),$$

where ν is sound velocity in the medium, β is volumetric thermal expansion coefficient, $\rho_{m}$ is mass density, $c_{p}$ is specific heat at constant pressure, k is thermal conductivity, $A_{e}$ is optical absorption coefficient at the excitation wavelength, I(r,t) is axially symmetric laser source intensity under pulsed excitation and $f=f_{\mathrm{em}}+f_{g}$ accounts for the body force densities inside the medium given by the electromagnetic force density $f_{\mathrm{em}}$ in addition to the gravitational force density $f_{g}$ in fluid media which can be neglected. Eq. (1) is valid for pressure fields of small amplitude propagating in inviscid fluids.

We would like to note that our approach of introducing electromagnetic force density into Eq. (1) differs from the classical description of the excitation of acoustic waves [18], [28]. For example, in anisotropic solids, the electromagnetic force density components $f_{\mathrm{em},i}$ as given in Eq. (1) can be replaced by $-\frac{\partial \sigma_{ij}}{\partial x_{j}}$, where $\sigma_{ij}$ are the mechanical stresses induced in the bulk of material by an external action such as with a laser pulse.

In Refs. [18], [28], the authors propose employment of the following equation for the mechanical stresses induced by electrostriction

(3) $$\sigma_{ij}=\frac{1}{8\pi }p_{klji}D_{k}D_{l}\;,$$

where $D_{i}$ are the components of the electric displacements vector inside the solid D and $p_{klji}$ are the photoelastic constants (elasto-optical coefficients or Brillouin scattering matrix elements). The components of the electric displacements field $D_{i}$ and the electric field $E_{j}$ are related by the dielectric tensor ${ɛ}_{ij}$ as $D_{i}={ɛ}_{ij}E_{j}$. This yields the following, traditionally invoked, form of the bulk force density due to electrostriction

(4) $$f_{\mathrm{em},i}=-\frac{\partial \sigma_{ij}}{\partial x_{j}}=-\frac{1}{8\pi }p_{klji}\frac{\partial \left({ɛ}_{km}{ɛ}_{ln}E_{m}E_{n}\right)}{\partial x_{j}}\;.$$

Returning back to our model, the source term for a pulsed Gaussian beam is given by

(5) $$I\left(r,t\right)=Q_{0}e^{-2r^{2}/w_{e}^{2}}f\left(t\right),$$

with the temporal profile of the pulsed excitation laser expressed by $f\left(t\right)=t_{0}^{-1}e^{-{\left(t-\xi \right)}^{2}/\tau^{2}}$, where the time of maximum irradiance and width are given by ξ and τ, respectively. Here, $t_{0}=\tau \sqrt{\pi }\left[1+\text{Erf}\left(\xi /\tau \right)\right]/2$ is a normalization parameter given in terms of the error function Erf(x), $Q_{0}=2E_{p}/\pi w_{e}^{2}$ is the heat source amplitude, $E_{p}$ and $w_{e}$ are the energy and radius of the excitation laser beam, respectively.

The force density $f_{\mathrm{em}}$ is given by the Microscopic Ampère formulation [23] as

(6) $$f_{\mathrm{em}}=-\frac{1}{2}{\left|E\right|}^{2}\nabla ɛ+\frac{1}{2}\nabla \left(P\cdot E\right)+\frac{n^{2}-1}{c^{2}}\frac{\partial \left(E\times H\right)}{\partial t}.$$

Here, E is the electric field, P is the polarization field, H is the magnetic field, ɛ and n are the sample electrical permittivity and refractive index, respectively, and c is the speed of light in vacuum. From our perspective, optical electrostriction is treated classically as the induced dipolar interactions with the optical field, and consists of the second term of Eq. (6) alone. It occurs in bulk and, for homogeneous media, cannot generate Brillouin scattering. This happens because the necessary spatial dependence generated in the index of refraction due to the thermal and pressure effects occur way after the electromagnetic pulse has left the medium. Therefore, our definition of electrostriction is different from the one associated to Eqs. (3), (4), where it is treated very generally as a quadratic effect in the displacement fields D. The first term in Eq. (6) is described as radiation pressure, and in our model occurs only at the interfaces. The last term is known as Abraham force and can be neglected.

By using Eqs. (5), (6), we obtain the optical electrostrictive force density as

(7) $$f_{\mathrm{em}}\left(r,t\right)=-F_{0}\;r\;e^{-2r^{2}/w_{e}^{2}}f\left(t\right)\overset{ˆ}{r},$$

where $F_{0}=16\left(n-1\right)E_{p}/\left[c\left(n+1\right)\pi w_{e}^{4}\right]$. The small effect of electrostriction in the direction of beam propagation has already been neglected. The sample is assumed to be sufficiently thick, so that the axial null thermal flux approximation can be applied and viscous surface effects can be neglected. In addition, the width of the excitation beam is much smaller than the lateral sample dimensions.

We report here on the semi-analytical solutions to Eqs. (1), (2) assuming low optical absorption and infinite radial boundary conditions. The first condition is valid for a large variety of fluids presenting $A_{e}L\ll 1$, where L is the thickness of the sample. At the nanosecond time scale, the latter approximation does not affect the thermal lens transients since the launched pressure wave only reaches the cuvette edge after the measurement terminates. Using the integral transform method, the solutions to the laser-induced thermal and pressure variations are given separately as

(8) $$T\left(r,t\right)=\frac{Q_{0}}{\rho_{m}c_{p}}\frac{1}{\left[1+\text{Erf}\left(\xi /\tau \right)\right]}\times \int_{0}^{\infty }\frac{w_{e}^{2}}{4}e^{-\frac{1}{8}w_{e}^{2}\alpha^{2}}\chi \left(D\alpha^{2},t\right)J_{0}\left(\alpha r\right)\alpha d\alpha$$

and

(9) $$p\left(r,t\right)=\int_{0}^{\infty }\frac{w_{e}^{2}}{4}e^{-\frac{1}{8}w_{e}^{2}\alpha^{2}}\;g\left(\alpha ,t\right)J_{0}\left(\alpha r\right)\alpha d\alpha ,$$

where $D=k/\left(\rho_{m}c_{p}\right)$ is the thermal diffusivity, $J_{0}$ is the 0th order Bessel function of the first kind and

(10) $$g\left(\alpha ,t\right)=\frac{Q_{0}}{c_{p}}\frac{\beta }{\tau }\frac{\nu }{\left[1+\text{Erf}\left(\xi /\tau \right)\right]}\frac{1}{2\alpha }\times \left(-\frac{4\sin\left(\nu t\alpha \right)}{\sqrt{\pi }}e^{-\xi^{2}/\tau^{2}}\right)+\left(\frac{}{}\nu \tau \alpha \left[\chi \left(i\nu \alpha \right)+\chi \left(-i\nu \alpha \right)\right]\right)+\frac{F_{0}w_{e}^{2}\left(i\nu \alpha \right)}{8\left[1+\text{Erf}\left(\xi /\tau \right)\right]}\left[\chi \left(-i\nu \alpha \right)-\chi \left(i\nu \alpha \right)\right],$$

in which

(11) $$\chi \left(x,t\right)=\left[\text{Erf}\left(\frac{\xi }{\tau }+\frac{x\tau }{2}\right)-\text{Erf}\left(\frac{\xi -t}{\tau }+\frac{x\tau }{2}\right)\right]\times e^{-x\left(t-\xi \right)+\frac{x^{2}\tau^{2}}{4}}.$$

The small variation Δn(r,t) of the sample’s local refractive index is expressed via the thermo-optic coefficient ∂n/∂T, the piezo-optic coefficient ∂n/∂p and the non-linear refractive index $n_{2}=\partial n/\partial I$ as the first terms in the series expansion, i.e., 

(12) $$\Delta n\left(r,t\right)=\frac{\partial n}{\partial T}T\left(r,t\right)+\frac{\partial n}{\partial p}p\left(r,t\right)+\frac{\partial n}{\partial I}I\left(r,t\right).$$

The three contributions generate the total phase shift of the probe beam, namely

(13) $$\Phi \left(r,t\right)=\frac{2\pi }{\lambda_{p}}\int_{0}^{L}\Delta n\left(r,t\right)\;\;dz,$$

where $\lambda_{p}$ is the probe beam wavelength in the sample. The first two terms are given by Eqs. (8), (9), while the last one is obtained directly from the spatiotemporal distribution of intensity in Eq. (5). Considering only the center of the probe beam spot at the detector plane in the far-field region and using Fresnel diffraction theory, the far-field intensity signal $S\left(t\right)$ can be written as [29]

(14) $$S\left(t\right)={\left|\int_{0}^{\infty }\frac{2r}{w_{p}^{2}}e^{-\left(1+iV\right)r^{2}/w_{p}^{2}-i\;\Phi \left(r,t\right)}dr\right|}^{2}\;\;,$$

where V is an experimental parameter and $w_{p}$ is the radius of the probe beam in the sample. The numerical calculation of $S\left(t\right)$ in Eq. (14) requires the determination of $T\left(r,t\right)$ and $p\left(r,t\right)$ considering all the effects of the radiation forces in the liquid, which is achieved by applying the semi-analytical solutions presented in Eqs. (8), (9).

## 3 Photo-induced lensing technique

**Fig. 1:**
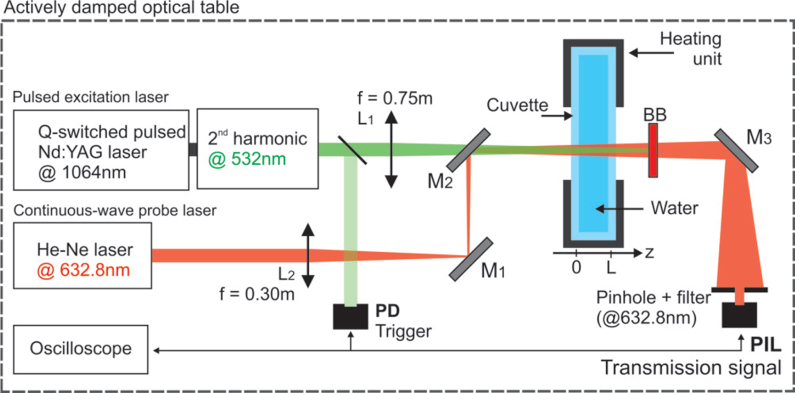
Photo-induced lensing (PIL) setup. Schematic of the time-dependent photo-induced lensing measurement setup. Green and red routes represent pump and probe laser beams, respectively. PD, M, L, and BB stand for photodetector, mirrors, lenses and beam block.

The time-resolved photo-induced lensing method used in this work is illustrated in Fig. 1 and a detailed description is given in Ref. [23]. A pulsed TEM_00_ laser operating at the low-absorption wavelength of 532nm with a FWHM pulse width of 9ns was used to excite a sample having thickness of L=5mm. The laser beam was focused in the sample using a lens with f=0.75 m focal length. A low-irradiance TEM_00_ He-Ne laser operating at 632.8nm wavelength travels almost collinearly (small angle of <0.5°) with the excitation beam and is focused by a lens (f=0.30 m) at 5 cm distance from the sample to probing the induced phase shift in the sample. The intensity variation of the probe beam center, after passing through the sample, was detected by a pinhole-laser line filter-photodetector assembly in the far field (5 m from the sample). The laser line filter is used to prevent the excitation laser beam and ambient light from being detected by a fast photodetector (200-MHz bandwidth). A digital oscilloscope recorded the signal. The excitation beam was used to trigger the oscilloscope by using another photodetector (same as the probe sensor) at a repetition frequency of 10Hz. The entire setup was aligned on precise, actively damped optical tables to eliminate mechanical vibration of the sample. A heating/cooling unit and a temperature controller were used to maintain the samples’ temperature at either $\left(4.0\pm 0.1\right)\;{}^{\circ}C$ or $\left(25.0\pm 0.1\right)\;{}^{\circ}C$. The excitation and probe beam radii were measured with a beam profile camera. The laser energy was measured using a pyroelectric energy sensor. The experimental parameter V=5.1. The uncertainties are smaller than 1% and correspond to the standard deviation of the mean over the course of experiments. A minimum of 15 measurements were performed for each sample with averaging performed over 512 transients to reduce noise.

## 4 Results and discussion

**Fig. 2:**
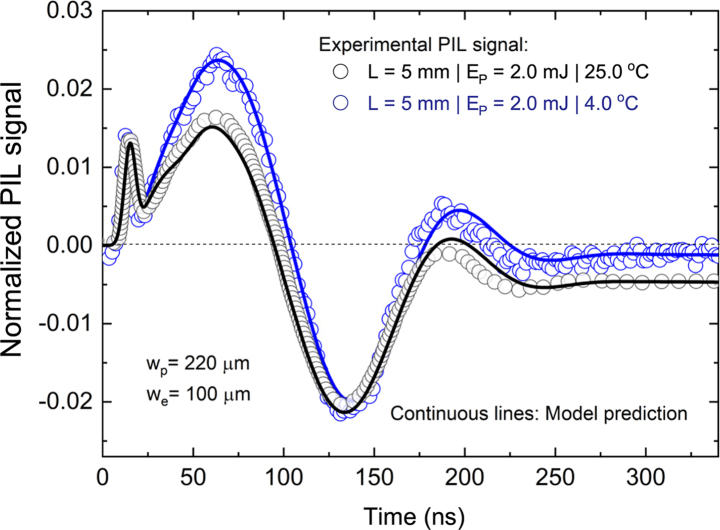
Time-dependent photo-induced lensing (PIL) transients. PIL signal under pulsed laser excitation at 532nm at different temperatures: $\left(4.0\pm 0.1\right)\;{}^{\circ}C$ and $\left(25.0\pm 0.1\right)\;{}^{\circ}C$. The transients show the optical power variation at the center of a continuous probe laser beam transmitted through the cuvette–water interfaces and measured by a photodetector in the far-field. Open symbols are experimental data while continuous lines represent the numerical calculations using the normalized signal $S\left(t\right)/S\left(0\right)-1$; confidence level of 95%. The uncertainties are smaller than 1% and correspond to the standard deviation of the mean over all the experiments.

Fig. 2 shows two averaged PIL transients for water (Milli-Q water) at $\left(4.0\pm 0.1\right)\;{}^{\circ}C$ and $\left(25.0\pm 0.1\right)\;{}^{\circ}C$. The time-dependent signal detected in the experiments shows only the center of the probe beam spot at the photodetector in the far-field region, and is normalized as $S\left(t\right)/S\left(0\right)-1$. Positive values of this signal correspond to the focusing effect of the perturbed volume with negative values denoting divergence. The experimental parameters are further listed in Fig. 2.

The optical Kerr effect (also dubbed as the AC Kerr effect) is well known to produce an intensity-dependent refractive index or non-linear index of refraction, $n_{2}$, that gives the rate at which the index changes with the laser intensity [14]. Both water and fused silica walls have a positive $n_{2}$ value of the same magnitude (see Table A.1) contributing to the phase shift induced to the probe beam. This effect follows the time dependence of the laser pulse, thus resulting in the first positive peak observed in the PIL transient signal at the very first 20 ns of the transient. The optical Kerr effect induces a short-lived Kerr lens with a focusing power due to the positive value of $n_{2}$. Focusing originates from the fact that the central part of the wavefront is delayed more than its lateral parts. In the presented experiments, the maximum focusing length of the Kerr lens occurring at the maximum optical power $P_{\max}$, equals to $\pi w_{e}^{4}/\left(8n_{2}LP_{\max}\right)$ = 6.5 m. This represents a simple and novel experimental approach to measure the value of $n_{2}$ for weakly absorbing liquids.

The transient signal that follows, from 20 ns to around 220 ns, is dominated by the launch of two types of elastic perturbations, namely, acoustic waves. The electrostriction-induced waves are given by $p_{\mathrm{es}}\left(r,t\right)$ and the thermoelastic ones by $p_{\mathrm{th}}\left(r,t\right)$. The two pressure fields add up linearly to produce the combined effect described by $p\left(r,t\right)=p_{\mathrm{es}}\left(r,t\right)+p_{\mathrm{th}}\left(r,t\right)$. Due to the problem’s linearity, Eq. (1) can thus also be solved individually yielding separate propagating pressure fields for each type of acoustic wave. There is no ambiguity on how to set the governing equation to properly predict $p_{\mathrm{th}}\left(r,t\right)$. However, until just recently the consensus has not been reached on what form of body force one should use to adequately describe acoustic waves generated via electrostriction at optical frequencies. It has just been experimentally demonstrated that the Microscopic Ampère force in Eq. (6) captures the experimental reality more accurately than other expressions for the radiation-type optical body force. The PIL signals (lines) in Fig. 2 have thus been calculated using the best known candidate for the optical body force, i.e., the Microscopic Ampère force density from Eq. (6), which for our Gaussian-type laser beam excitation results in Eq. (7). Here the contribution from the cuvette walls to the PIL signal have been neglected as it accounts for less than 1% of the total signal. Note that the numerical predictions are in excellent agreement with the experimental data. The propagation of acoustic waves dispersed in water can be calculated using Eq. (9).

The fourth contribution to the PIL signal, termed the thermal lens, is clearly seen from 220 ns on when the two types of acoustic waves simultaneously exit the volume probed by the continuous He-Ne laser. This signal offset occurs when optical energy is deposited as heat during the time-of-flight of the excitation laser pulse and decays on a much longer scale than the experimental temporal window of interest.

**Fig. 3:**
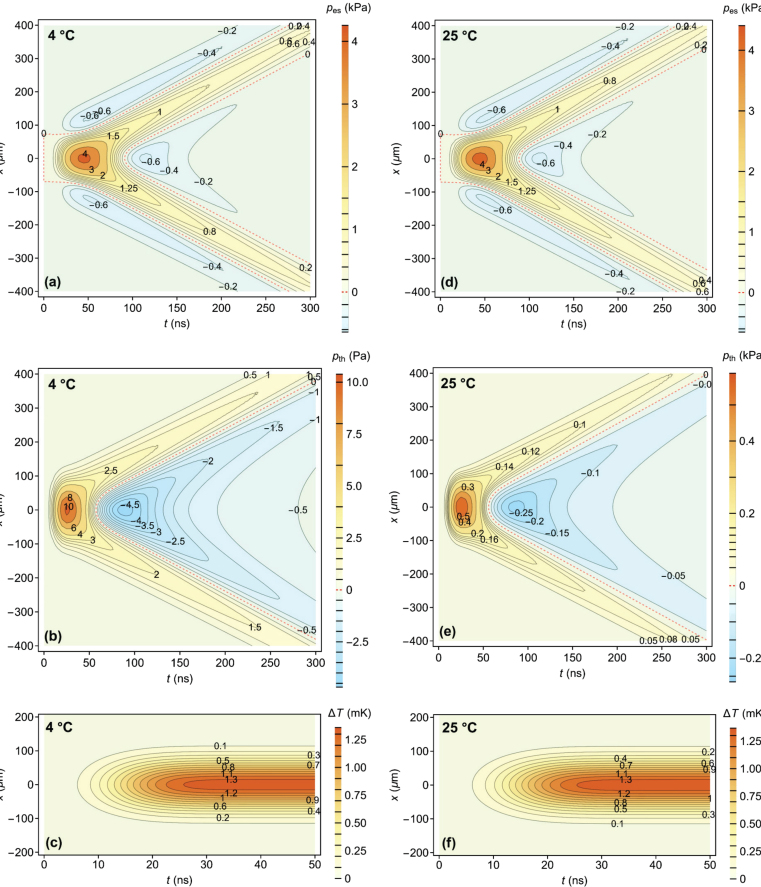
Pressure and temperature distributions. Spatiotemporal pressure distribution within the cross-section of the sample due to (a,d) electrostriction force [$p_{\mathrm{es}}\left(x,t\right)$] and (b,e) heat deposition [$p_{\mathrm{th}}\left(x,t\right)$]. (c,f) The temperature distribution [T(x,t)] in the sample. Left column (a)–(c) shows the results for 4.0 °C and right column (d)–(f) for 25.0 °C. Note the different scales: Pa in (b) and kPa in (e).

To better understand the measured PIL signals presented in Fig. 2, we further display the calculated cross-sections of the time-dependent pressure distributions in water separately for the one caused by electrostriction, $p_{\mathrm{es}}\left(x,t\right)$ [Fig. 3(a) and (d)], and by heat deposition, $p_{\mathrm{th}}\left(x,t\right)$ [Fig. 3(b) and (e)], at the temperature 4.0 °C [Fig. 3(a)–(c)] and at 25.0 °C [Fig. 3(d)–(f)]. Similarly, the temporal evolution, T(x,t), of the temperature field is given in Fig. 3(c) and (f). The Cartesian coordinate used to display the spatial domain of the cross-section is denoted by x. The time of maximum irradiance and width are ξ=20ns and τ=9ns, respectively. The energy of the pulse is $E_{p}=\;2.0\;\mathrm{mJ}$ and excitation beam radius is $w_{e}=\;\mathrm{100}\;\mu m$. The parameters used for the calculations are presented in Table A.1.

The following discussion illuminates the difference between the two PIL transients and the corresponding pressure and temperature fields at two chosen temperatures. The Kerr lens (dominant at 0 ns–20 ns) is theoretically identical at either 4.0 °C or 25.0 °C. The only material parameter this lens-type effect depends on is the change of the refractive index with the intensity of the excitation light. The value is the same for both temperatures studied (see also Table A.1) and does not depend on the induced temperature and pressure fields.

The thermal lens contribution to the PIL signal (dominant from 220 ns on, but constant already from 30 ns on) is directly dependent on the optical absorption coefficient of water (approximately $0.045\;m^{-1}$ at 532 nm at both temperatures studied). Although very small, it causes a temperature increase of almost 1.4 mK along the axial dimension, as shown in Fig. 3(c) and (f). This small temperature change is practically indiscernible at both temperatures studied. However, when combined with $\partial n/\partial T=-15\times 10^{-6}$ $K^{-1}$ at 4.0 °C and $\partial n/\partial T=-96\times 10^{-6}$ $K^{-1}$ at 25.0 °C, its effect on the PIL transient differs significantly. After the acoustic waves exit the probing volume, this phenomenon dictates the PIL quasi-steady state signals. Due to a negative value of ∂n/∂T, the persisting thermal lens acts as a divergent lens, generating a negative contribution to the PIL signal. The ratio of ∂n/∂T values for both temperatures (i.e., 96/15 = 6.4) directly reflects the observed DC offset difference in the PIL signal from 220 ns on, as the quasi-steady state PIL signal is significantly closer to zero signal at 4.0 °C compared to 25.0 °C. Since the optical absorption coefficient of fused silica is lower by as much as one order of magnitude as compared to that of water, the thermal lensing in the cuvette walls can be safely neglected.

The pressure waves caused by the radiation force (electrostriction) and by heat-deposition-induced pressure gradient propagate laterally from the excitation volume within the samples. When present within the probed volume, these effects dominate over the thermal lens signal. Of the two acoustic waves, the one induced via electrostriction has an amplitude larger than the thermoelastic one at 25.0 °C and this difference is even larger at 4.0 °C. In fact, the effect of radiation forces was only perceived in the experiments due to a perfect combination of thermal, optical, and acoustic properties of the samples. For both temperatures, the electrostrictive force compresses the liquid, and the time required for pressure equilibrium to be established in water is on the same order as the time needed for sound to traverse the probed volume (with radius $w_{p}$). Due to the finite excitation beam width $w_{e}$, the acoustic waves require $\left(w_{p}+w_{e}\right)/\nu \approx 220\;\mathrm{ns}$ to travel over this distance, which is in agreement with the experimental results shown in Fig. 2.

Note that at 4.0 °C the temperature derivative of the mass density of water nears zero (see the value of β in Table A.1), which results in a very small pressure gradient due to heat deposition. This is clearly seen when comparing Fig. 3(b) and (e), where the ratio of the pressure maxima is (588 Pa)/(10.4 Pa) = 57, reflecting the ratio of the volumetric expansion coefficient (261 K^−1^)/(5 K^−1^) = 52 at the two temperatures studied.

As discussed in Section 2, in our model the pressure and temperature variations do not induce stimulated Brillouin scattering because they both are developed only after the pulsed laser source has left the sample. The change in n due to the optical Kerr effect, on the other hand, occurs at the same characteristic time of the pulse width, and therefore should generate an extra contribution to the local electromagnetic force density. This contribution, however, is negligible since the maximum relative variation in n due to this effect is of order $n_{2}I_{\max}\approx 10^{-10}$. The radiation pressure acting at the interfaces between water–glass and glass–air is in our experimental configuration also found to be negligible (by orders of magnitude) as compared to the optical electrostriction term. This is so, because in our work the probing geometry is highly sensitive to the radial refractive index gradients generated in the volume by the electrostriction effect and is insensitive to the axial gradients.

Theoretical treatments of optoacoustic source generation by simultaneously accounting for thermal expansion and electrostriction [17], [30], [31], [32], [33] have revealed two important times scales: the temporal duration of the excitation pulse ($\tau_{p}∼10\;\text{ns}$ in the current work) and the acoustic transit time ($\tau_{a}=w_{e}/\nu ∼68\;\text{ns}$) across the excitation beam radius. When $\tau_{p}<\tau_{a}$, as in our experiments, the spatial extent of the pulse plays a more significant role than its temporal span. This is known as a wide (broad) beam case of a line (cylindrical) optoacoustic source [31], [34], [35]. As expected, the pressure amplitude of both types of elastic waves decays as $r^{-1/2}$, which is a characteristic decay rule for acoustic waves emitted from a line source. As already described, the acoustic pressure p can be written as the sum of a thermal expansion term $p_{\mathrm{th}}$ and an electrostriction term $p_{\mathrm{es}}$, the latter being proportional to the time derivative of $p_{\mathrm{th}}$. Hence, whenever $p_{\mathrm{th}}$ reaches its peak, $p_{\mathrm{es}}$ crosses the zero value [34], [36], [37]. This also appears to be true for the thermal and the electrostrictive parts of the calculated pressure shown in Fig. 3(a) and (b) at 4.0 °C or Fig. 3(d) and (e) at 25.0 °C. Note the coincidence of the zero pressure in $p_{\mathrm{th}}\left(x,t\right)$ with the pressure extreme in $p_{\mathrm{es}}\left(x,t\right)$.

To sum it up, the main difference in the PIL transients between 4.0 °C and 25.0 °C originates from the large differences in ∂n/∂T and β, both heavily dependent on the temperature. The former material property of water affects the thermal lens and the latter the emission of thermoelastic waves. In general, the overall PIL signal is jointly contributed by all these distinct effects occurring simultaneously in the sample. All the forces, in addition to the optical Kerr effect occurring during the pulse duration, represent the total experimental PIL signal observed in the experiments. All these effects are linearly dependent on the optical path length and excitation pulse energy (not shown here), and so are the experimental signals observed when changing the temperature, as displayed in Fig. 2. Evidently, the numerical calculations using the Microscopic Ampère formulation in Eq. (7) are in excellent agreement with our experimental results.

## 5 Conclusions

In summary, we have shown that experiments can be constructed in a way that electrostriction plays the dominant role in acoustic wave generation by transient illumination of a weakly absorbing liquid phase. The effect is accompanied by acoustic waves generated by heat-deposition and the thermal lens. In addition, optical Kerr effect also takes place during laser excitation. By employing the unique thermal property of water at 4.0 °C, we have further demonstrated that both the thermal lens effect and the thermoelastic waves can be further suppressed, exposing electrostriction-generated acoustic waves as the sole disturbance affecting the optical photo-induced lensing signal. We have also presented a new semi-analytical model to numerically calculate the temperature and pressure distributions as functions of space and time in low-absorbing fluid media under optical excitation. It was quantitatively found to be in good agreement with the experiments performed in water. Additional temporal separation of the Kerr lens signal from the electrostriction-launched acoustic waves gives access to a new experimental approach for measuring the non-linear refractive index $n_{2}$.

## CRediT authorship contribution statement

**N.G.C. Astrath:** Project administration, Conceptualization, Formal analysis, Investigation, Writing – original draft, Writing – review & editing. **B. Anghinoni:** Investigation, Writing – review & editing. **G.A.S. Flizikowski:** Investigation. **V.S. Zanuto:** Investigation. **L.C. Malacarne:** Investigation. **M.L. Baesso:** Investigation, Writing – review & editing. **T. Požar:** Investigation, Writing – review & editing. **D. Razansky:** Investigation, Writing – review & editing.

## Declaration of Competing Interest

The authors declare that they have no known competing financial interests or personal relationships that could have appeared to influence the work reported in this paper.

## Figures and Tables

**Table A.1:** Physical properties used in the simulations [29], [38].

Parameter	Units	Water	Water	Fused silica
		25 ${}^{\circ }C$	4 ${}^{\circ }C$	$∼25{\;}^{\circ }C$
∂n/∂p[39]	$10^{-10}\;{\mathrm{Pa}}^{-1}$	1.40	1.68	0.031
∂n/∂T	$10^{-6}\;K^{-1}$	−96	−15	−9.6
ν	$m\;s^{-1}$	1481	1423	5800
$n_{2}$	$10^{-20}\;m^{2}\;W^{-1}$	1.0	1.0	1.7
$A_{e}$[40]	$m^{-1}$	0.045	0.045	0.005
β	$10^{-6}\;K^{-1}$	261	5	0.55
$c_{p}$	$J\;{\mathrm{kg}}^{-1}\;K^{-1}$	4182	4206	703
k	$W\;m^{-1}\;K^{-1}$	0.602	0.564	1.38
$\rho_{m}$	$\mathrm{kg}\;m^{-3}$	998	999	2203

## Data Availability

Data will be made available on request.

## Appendix Physical properties used in the simulations

The physical parameters of water at 25 °C and 4 °C are presented in Table A.1.
